# Process-Compatible Immobilization of Thermolabile Enzymes for Self-Degradable Polyesters via Industrial Melt Compounding Conditions

**DOI:** 10.3390/polym18151830

**Published:** 2026-07-26

**Authors:** Jeong Je Seo, In-Woo Nam, HyoJung Kim, Hajoong Kim, Jung Nam Im, Hyun Ju Oh, Jungyeon Kim, Byoung-Sun Lee, Yeong Og Choi

**Affiliations:** 1 Textile Innovation R&D Department, Korea Institute of Industrial Technology, Ansan 15588, Republic of Korea; tjwjdwp12@naver.com (J.J.S.); iwnam79@kitech.re.kr (I.-W.N.); kim00502@naver.com (H.K.); founder@kitech.re.kr (J.N.I.); hjoh33@kitech.re.kr (H.J.O.); cobalt98@kitech.re.kr (J.K.); 2 Department of Fiber System Engineering, Dankook University, Yongin 16890, Republic of Korea; gkwnd98@naver.com

**Keywords:** enzyme immobilization, thermally stable biocatalysts, bio-based circular polymers, industrial composting, controlled biodegradation

## Abstract

Integrating thermolabile enzymes into industrial melt processing remains a key challenge for achieving programmable self-degradable plastics. Here, we demonstrate a process-compatible stabilization strategy for bio-based polyesters using immobilized Alcalase. Two approaches were compared: adsorption onto zeolite (Z-En) and entrapment within a citric acid–crosslinked carboxymethyl cellulose matrix (C-En-CA). Poly(lactic acid) (PLA) masterbatches containing 10 wt% C-En-CA retained catalytic functionality after twin-screw extrusion at temperatures up to 210 °C. Hydrolytic testing in 0.05 M Tris–HCl buffer (pH 8.0) resulted in a 10.03% mass loss after 3 weeks, confirming enzyme survival following melt compounding. When incorporated into PBAT T-die films, the C-En-CA system achieved 79.5% biodegradation within 45 days under industrial composting conditions. These results demonstrate that appropriate immobilization enables enzymatic stabilization under realistic extrusion temperatures, offering a scalable pathway toward controllable end-of-life degradation in commercially relevant biodegradable plastics. Ultimately, this study establishes a new paradigm for polymer–enzyme composites by overcoming the long-standing 200 °C thermal barrier, effectively unlocking the practical deployment of biocatalytic masterbatches in industrial manufacturing.

## 1. Introduction

The growing environmental burden of persistent petroleum-based plastics and proliferation of microplastics, recognized as critical threats to global ecosystems, have accelerated the development of biodegradable polymer alternatives [1,2,3]. Aliphatic polyesters such as poly(lactic acid) (PLA) and poly(butylene adipate-co-terephthalate) (PBAT) are among the most commercially relevant candidates owing to their compostability and compatibility with conventional thermoplastic processing [4,5,6]. PLA, derived from renewable feedstocks, provides high stiffness and transparency [7], whereas PBAT offers flexibility and toughness, making their blends particularly attractive for film and packaging applications [8]. The global market for PLA and PBAT is projected to reach approximately USD 1.5 billion by 2025, with compound annual growth rates of 16.3% and 12.8%, respectively [9,10]. Despite these advantages, biodegradable polymers exhibit inherent limitations. Their degradation typically requires industrial composting conditions [11], and degradation rates under ambient environments are often slow or poorly controlled [12]. Resolving the durability-degradability paradox, maintaining integrity during service while ensuring predictable end-of-life degradation, remains a central challenge in sustainable polymer engineering [13].

**Figure 1:**
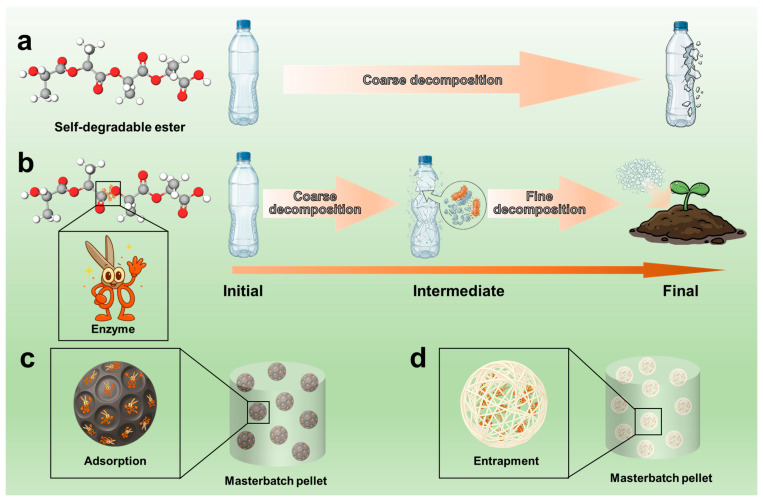
Comparison of plastic degradation pathways and enzyme integration strategies. (**a**) Traditional abiotic coarse decomposition. (**b**) Sequential biocatalytic degradation involving initial, intermediate, and final stages of mineralization. Schematic representation of the engineered masterbatch pellets incorporating enzymes via (**c**) physical adsorption and (**d**) matrix entrapment.

Self-degradable polyesters such as PLA and PBAT have attracted considerable attention as sustainable alternatives to petroleum-based plastics because of their tunable physicochemical properties and potential for biological end-of-life management [14,15]. Their appeal largely arises from their ability to degrade into environmentally benign products, including water, carbon dioxide, and biomass, through microbial activity under composting, soil, or marine conditions [16,17]. However, the degradation rate strongly depends on intrinsic polymer characteristics, such as molecular weight, crystallinity, and chemical composition, as well as extrinsic factors including geometry, surface area, and environmental conditions. In practice, both PLA and PBAT often require elevated temperatures and sufficient enzymatic activity to achieve rapid decomposition [18]. Under ambient conditions, neat self-degradable polyesters typically undergo only slow surface erosion and some fragmentation into coarse pieces, resulting in prolonged degradation periods (Figure 1a).

To overcome these limitations, Enzyme-mediated hydrolysis provides a selective and environmentally benign route to accelerate polyester degradation [19]. Protease- and esterase-type enzymes can catalyze chain scission under mild aqueous conditions, enabling controlled depolymerization without harsh chemicals [20]. In the presence of an adequate concentration of active enzymes, it can significantly accelerate hydrolytic chain scission, promoting fragmentation into coarse pieces and ultimately conversion into fine biomass (Figure 1b). Therefore, incorporating enzymes directly into polyester matrices represents a promising strategy to achieve controlled and accelerated self-degradation. Among various candidates, Alcalase was selected in this work as an efficient biocatalyst to enhance polyester decomposition [21]. Alcalase, produced by Bacillus licheniformis, is a serine protease containing a catalytic triad in which the hydroxyl (–OH) group of serine and the imidazole ring of histidine play central roles in ester and peptide bond cleavage. In addition, the enzyme backbone and side chains contain amide (–CONH–), amino (–NH_2_/–NH_3_^+^), and carboxylate (–COO–) groups that contribute to its polarity and intermolecular interactions.

The enzymatic decomposition of polyesters proceeds via a well-established serine hydrolase mechanism involving four key steps:

(i) Nucleophilic attack on the carbonyl (C=O) carbon of the ester bond by the serine-derived alkoxide, generated through deprotonation of the serine hydroxyl by histidine;
(ii) Formation of an acyl–enzyme tetrahedral intermediate, followed by protonation of the leaving group by histidine and cleavage of one end of the ester bond;
(iii) Nucleophilic attack on the carbonyl carbon of the acyl–enzyme intermediate by a water molecule, accompanied by histidine-mediated proton transfer to generate a hydroxide species;
(iv) Collapse of the second tetrahedral intermediate, leading to enzyme regeneration and release of the hydrolyzed product [22].

Despite this efficient catalytic pathway, the practical implementation of enzyme–polymer composites is severely limited by the intrinsic thermal sensitivity of enzymes during melt processing [18]. Direct incorporation of free enzymes into thermoplastic matrices is impractical because most enzymes denature at temperatures far below typical melt-processing conditions (approximately 180–200 °C) [23,24]. Thus, immobilization strategies including adsorption, entrapment, and encapsulation have been widely explored to confer conformational rigidity [25,26,27,28,29]. Physical adsorption offers simplicity and scalability but risks enzyme leaching [30], whereas polymeric entrapment provides mechanical shielding at the cost of mass-transfer resistance, and encapsulation maintains transport while preventing leaching but demands complex, costly preparation [31,32,33,34]. Previous studies on enzyme-assisted biodegradable plastics have largely relied on low-temperature blending [35], solution casting [36], surface coating [37], or additional processing treatments such as hydrothermal pretreatment [38]. While these approaches demonstrated accelerated degradation, they often lacked industrial scalability or suffered from heterogeneous dispersion and partial enzyme deactivation. Reports of enzyme survival during melt compounding are largely limited to low-melting polymers such as poly(butylene succinate) (PBS), polycaprolactone (PCL), and poly(butylene succinate-co-adipate) (PBSA), processed at relatively low temperatures (≤130 °C) [39,40]. Poly(propylene succinate) (PPS) and calcium ion–bound carboxymethyl cellulose (CaCMC) have been utilized to preserve enzyme activity at temperatures up to 150 °C [41]. However, systematic evaluations of immobilization strategies remain scarce under the realistic extrusion temperatures required for PLA and PBAT, which typically exceed 150 °C. Consequently, there remains a critical need for thermally stabilized biocatalytic systems capable of withstanding melt processing while retaining sufficient activity to enable programmable self-degradation.

In this work, we present a dual immobilization strategy to integrate thermally stabilized enzymes into melt-processed biodegradable polyesters with a high processing temperature ≥ 170 °C. As schematically illustrated in Figure 1c,d, Alcalase was immobilized via adsorption within porous zeolite frameworks (Z-En) and via entrapment in a citric acid–cross-linked carboxymethyl cellulose matrix (C-En-CA). The resulting complexes were compounded into PLA masterbatches under industrially relevant extrusion temperatures and subsequently incorporated into PBAT films. This approach enables preservation of enzymatic functionality after high-temperature processing and allows programmable hydrolytic and composting degradation without compromising film processability. By coupling immobilization chemistry with scalable melt processing, this study establishes a practical pathway toward controllable and application-oriented biodegradable polymer systems. To the best of our knowledge, this study successfully addresses a major technical bottleneck in the field by demonstrating the industrial feasibility of melt-compounding thermolabile enzymes at temperatures above 200 °C, a milestone that has remained unreached to date [42].

## 2. Results and Discussion

**Figure 2:**
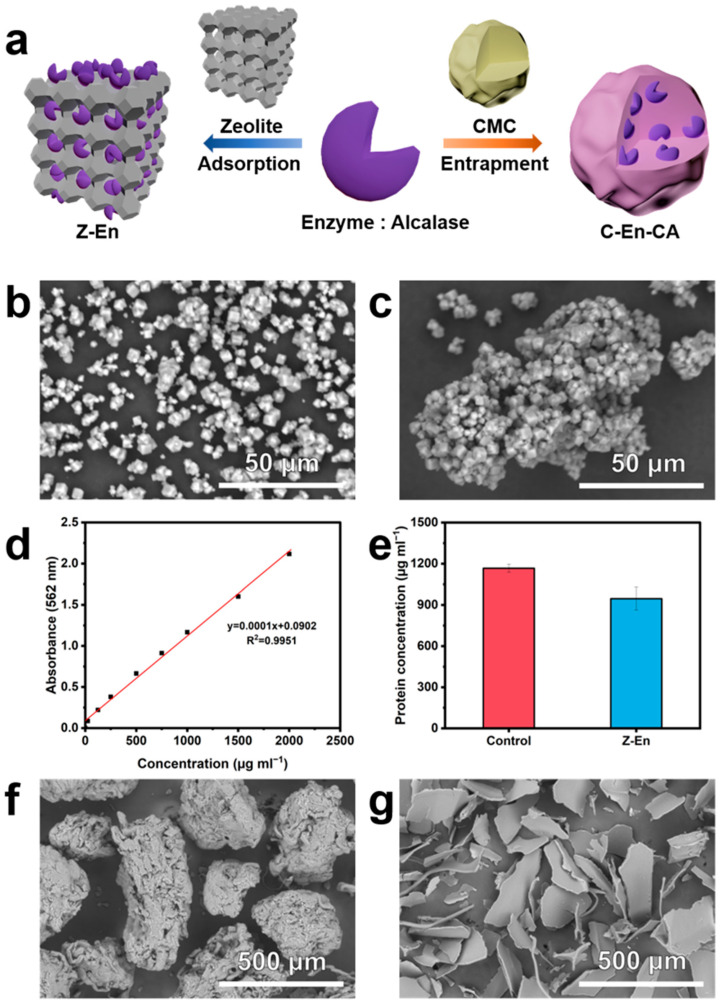
Immobilization of Alcalase on zeolite (Z-En) and carboxymethyl cellulose (C-En-CA) scaffolds. (**a**) Schematic illustration of the two immobilization strategies: surface adsorption onto zeolite (Z-En) and entrapment within a carboxymethyl cellulose matrix (C-En). (**b**,**c**) SEM micrographs of pristine zeolite and Z-En, respectively; the increased agglomeration in (**c**) suggests enzyme-mediated cohesive forces between particles. (**d**) BCA protein assay calibration curve (0–2000 µg mL^−1^) used for quantification. (**e**) Comparison of initial (Control) and residual (Z-En) protein concentrations in the supernatant, indicating a calculated immobilization efficiency of approximately 16.60%. (**f**,**g**) SEM micrographs of pristine CMC and the citric acid–crosslinked C-En-CA complex; the transformation from random particles to high-aspect-ratio sheet/flake structures in (**g**) highlights the morphological shift following enzyme entrapment and freeze-drying.

The immobilization approaches, adsorption onto zeolite (denoted as Z-En) and citric acid–crosslinked carboxymethyl cellulose matrix (denoted as C-En-CA), are illustrated in Figure 2a. The distinct performance difference between the two systems arises from their fundamental immobilization principles and the resulting differences in structural stability during harsh processing. Specifically, the Z-En system relies on physical adsorption, where the enzyme is attached to the surface of the porous inorganic support primarily through electrostatic interactions, hydrogen bonding, and hydrophobic interactions [43]. Although this adsorption method is highly advantageous for large-scale production due to its procedural simplicity and cost-effectiveness, it possesses an inherent structural limitation because the weakly bound enzymes can easily desorb from the host surface under external environmental fluctuations and high shear forces.

In contrast, the C-En-CA system utilizes a three-dimensional entrapment approach where the CMC matrix forms a robust, chemically crosslinked network via citric acid [44], mechanically cage-locking the enzyme molecules inside. This steric confinement shields the enzyme far more effectively from external thermal and mechanical stresses encountered during melt processing. At the molecular level, Alcalase is stabilized during melt processing through two key synergistic mechanisms. First, dehydration-induced structural rigidity plays a vital role; lyophilization fully removes water molecules, which severely restricts the conformational flexibility of the enzyme backbone. In this rigid, anhydrous state, the protein is maintained in a reversibly inactivated form, intrinsically suppressing irreversible thermal denaturation [45]. Second, a high-density hydrogen bonding network is formed simultaneously because the abundant carboxyl (–COOH) and hydroxyl (–OH) functional groups present within the crosslinked CMC–citric acid network establish multi-point hydrogen bonds with the amino acid residues of the enzyme [46]. This dense intermolecular network physically anchors the enzyme structure, preventing it from unfolding and maintaining the integrity of the catalytic active sites even under high temperatures up to 210 °C and intense shear forces.

Initially discrete zeolite particles observed in the SEM image (Figure 2b) became noticeably agglomerated after the enzyme adsorption process (Figure 2c). This agglomeration is attributed to enhanced interparticle cohesive forces arising from enzyme-mediated surface interactions. Importantly, the primary morphology of the individual zeolite particles remained unchanged, indicating that adsorption occurred predominantly at the surface without structural collapse of the inorganic scaffold. The immobilization efficiency of Z-En was quantitatively evaluated using a bicinchoninic acid (BCA) protein assay from 0 to 2000 µg mL^−1^ (Figure 2d). As shown in Figure 2e, the immobilization efficiency of the Z-En was approximately 16.60%, calculated from the difference between the initial protein concentration (C_0_ = 1166.8 µg mL^−1^) and the supernatant concentration after adsorption (C_s_ = 973.1 µg mL^−1^).

In contrast, the randomly shaped carboxymethyl cellulose (CMC) particles shown in Figure 2f underwent a pronounced morphological transformation after enzyme entrapment and subsequent freeze-drying for water removal (Figure 2g). Citric acid (CA, Sigma-Aldrich, St. Louis, MO, USA), an eco-friendly crosslinking agent [47], was employed to enhance the dimensional stability of CMC by preventing moisture-induced deformation in the CMC immobilizing system (denoted as C-En-CA). The resulting sheet- or flake-like structures provide a substantially increased surface-to-volume ratio. Such a two-dimensional architecture is advantageous for enhancing interfacial contact between the C-En-CA and the polyester matrix, thereby improving dispersion stability during melt processing and promoting greater enzyme accessibility to the polymer chains during degradation.

**Figure 3:**
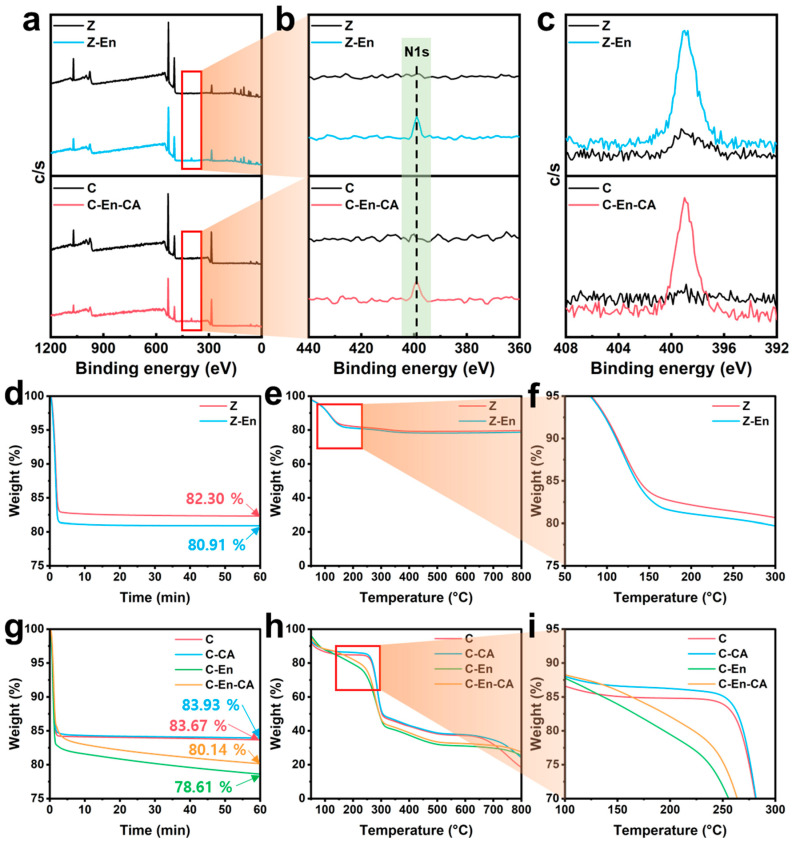
Chemical composition and thermal stability analysis of immobilized Alcalase. (**a**–**c**) XPS analysis for nitrogen identification: (**a**) Wide-scan survey spectra and (**b**) magnified N 1s regions showing the appearance of a nitrogen peak at ~400 eV upon enzyme immobilization; (**c**) High-resolution N 1s spectra centered at 399.5 eV, confirming the presence of imidazole groups from the Alcalase protein structure. (**d**–**f**) Thermal characterization of Zeolite-based complexes: (**d**) Isothermal TGA curves at 200 °C showing a 1.39% weight difference between Z and Z-En due to enzyme decomposition; (**e**,**f**) Dynamic TGA curves and their magnified regions (50–300 °C) confirming moisture desorption and the onset of enzyme degradation. (**g**–**i**) Thermal characterization of CMC-based complexes: (**g**) Isothermal TGA at 200 °C comparing C, C-CA, C-En, and C-En-CA, highlighting a 3.79% Alcalase loading and enhanced stability via entrapment; (**h**,**i**) Dynamic TGA curves demonstrating that citric acid (CA) crosslinking and enzyme entrapment stabilize the matrix up to the major degradation point (~250 °C).

To confirm the enzyme immobilization and uniformity, X-ray photoelectron spectroscopy (XPS) and energy-dispersive X-ray spectroscopy (EDS) elemental mapping were performed on both the zeolite (Z-En) and the crosslinked carboxymethyl cellulose (C-En-CA) scaffolds. Table S1 summarizes the surface atomic compositions of the Z, Z-En, C, and C-En-CA samples. Upon the immobilization of Alcalase, the carbon and nitrogen contents remarkably increased, whereas those of the other framework elements decreased. This quantitative shift clearly confirms the successful grafting and presence of the enzyme molecules on both matrices. Because the pristine zeolite and CMC carriers do not contain nitrogen, whereas the protein structure of Alcalase is rich in nitrogen from its peptide backbones and amino acid residues, nitrogen serves as an ideal elemental marker for the immobilized enzyme. In the wide-scan XPS spectra (Figure 3a), a distinct new peak corresponding to N 1s appeared at approximately 400 eV upon the introduction of Alcalase [48]. The high-resolution XPS spectra (Figure 3c) reveal a single, broad N 1s peak centered at approximately 399.5 eV for both Z-En and C-En-CA, which is attributed to the enzyme-derived nitrogen. In a more detailed manner, the main contribution to this broad envelope is associated with the peptide amide groups (N–C=O) typically observed around 399.5 eV. Meanwhile, minor contributions are expected to arise from the protonated amino groups (NH_3_^+^) of the amino acid residues at higher binding energies (~401.8 eV) and the aromatic nitrogen (C=N) of the histidine imidazole ring at lower binding energies (~398.3 eV) [49]. Therefore, the N 1s signal comprehensively represents the successfully immobilized protein backbone and its constituent amino acid side chains.

Furthermore, as shown in Figure S1, the enzyme-specific calcium (Ca) signal was uniformly distributed across and within the respective matrices, demonstrating homogenous enzyme loading. While transmission electron microscopy (TEM) was employed to visualize individual enzyme domains at higher magnification, a clear differentiation between the organic enzyme and the polymeric/amorphous matrix phases was hindered by insufficient electron contrast. This lack of imaging contrast is consistent with previous literature on Alcalase immobilization, wherein the low phase and crystallinity contrast between the organic biomolecules and the host carrier typically precludes direct TEM visualization in the absence of high-density nanoparticle labeling [50]. Consequently, exploring alternative high-resolution labeling techniques remains a focus for our future methodology optimization.

Quantitative and qualitative evaluations of Alcalase content in Z-En and C-En were conducted to confirm successful enzyme immobilization. First, isothermal weight change was monitored using a thermogravimetric analyzer (TGA) at 200 °C after rapid heating at 100 °C min^−1^. As described above, Alcalase contains hydroxyl (–OH), amide (–CONH–), amino (–NH_2_/–NH_3_^+^), and carboxylate (–COO^−^), groups, which undergo thermal decomposition at temperatures ≥ 200 °C [51,52,53,54]. Although the temperature was maintained at 200 °C, rapid mass loss was observed within the first 3 min, followed by gradual weight loss thereafter. The majority of weight loss in zeolite (denoted as Z, 17.70%) corresponds to moisture desorption [55]. In contrast, Z-En exhibited a greater total weight loss (19.09%) (Figure 3d), attributable to the additional contribution from adsorbed Alcalase. The difference in final weight (1.39%) corresponds to the immobilized Alcalase content, which was recalculated as 1.72% relative to the dry zeolite weight after excluding water content. Dynamic TGA results show that thermal decomposition of the enzyme-containing sample initiated at approximately 50 °C (Figure 3e) and was largely completed by around 150 °C (Figure 3f). The additional weight loss observed for Z-En compared to Z further confirms Alcalase adsorption.

Meanwhile, the thermal properties of CMC and enzyme-loaded CMC were investigated using both isothermal and dynamic TGA. The effects of citric acid (CA) crosslinking and enzyme immobilization were examined by comparing raw CMC (C), CA-crosslinked CMC (C-CA), Alcalase-loaded CMC (C-En), and CMC containing both CA and Alcalase (C-En-CA). Samples without Alcalase exhibited stable weight retention after rapid initial weight loss within 2 min, with a minor weight difference of 0.26% corresponding to CA content. In contrast, Alcalase-containing samples showed continuous weight loss up to 60 min due to the gradual thermal decomposition of the enzyme, suggesting enhanced thermal stability of Alcalase when entrapped within the CMC matrix (Figure 3g). This sustained resistance to rapid thermal collapse confirms that the steric hindrance and high-density hydrogen bonding within the crosslinked CMC matrix successfully protect the entrapped Alcalase at commercial processing thresholds. Furthermore, the weight difference between C-CA and C-En-CA was 3.79%, which is more than twice the Alcalase content observed in Z-En.

The dynamic TGA curves of CMC-based samples exhibited trends consistent with the isothermal results. The influence of Alcalase incorporation was noticeable from 150 °C up to 250 °C (Figure 3h,i), just prior to the major thermal degradation of CMC [56]. This substantial increase in the onset temperature (from ~50 °C to ~150 °C) demonstrates that the C-En-CA matrix provides an exceptional thermal shielding effect. Furthermore, this gradual and broad thermal decomposition behavior is highly beneficial for high-temperature polymer processing. Notably, CA crosslinking did not significantly affect thermal stability up to 650 °C. Above this temperature, weight loss was suppressed to a greater extent in samples without Alcalase (above 650 °C) and in those with Alcalase (above 750 °C), indicating structural stabilization effects. Consequently, these findings confirm that Alcalase was successfully adsorbed onto zeolite and effectively entrapped within the CMC matrix, with the entrapment strategy significantly enhancing both the immobilized enzyme content and its thermal stability.

**Figure 4:**
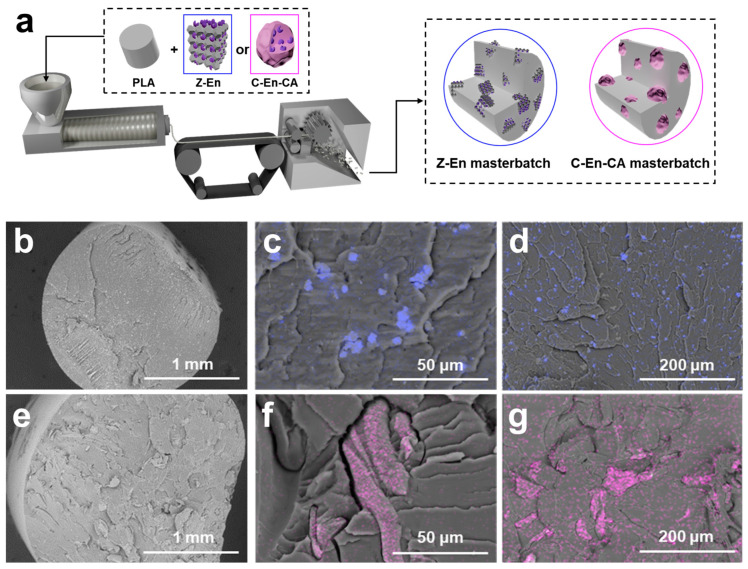
Fabrication and cross-sectional morphology of Alcalase-immobilized PLA masterbatches. (**a**) Schematic representation of the melt-extrusion and pelletization process using a twin-screw extruder to incorporate Z-En and C-En-CA into the PLA matrix. (**b**–**d**) Morphology and elemental distribution of Z-En/PLA: (**b**) Cross-sectional SEM image showing relatively round domains of ceramic-based Z-En; (**c**,**d**) High- and low-magnification EDS mapping of Aluminum (Al, blue), confirming the uniform dispersion of micron-sized zeolite particles. (**e**–**g**) Morphology and elemental distribution of C-En-CA/PLA: (**e**) SEM image showing increased pellet eccentricity and larger internal grains; (**f**,**g**) High- and low-magnification EDS mapping of Sodium (Na, purple), highlighting the formation of characteristic sheet-like CMC domains (tens of micrometers in size) within the polymer matrix.

Based on the enhanced thermal stability of immobilized Alcalase, Z-En and C-En-CA were incorporated into a PLA masterbatch using a twin-screw extruder at temperatures above the melting point of PLA (170–210 °C), followed by pelletization (Figure 4a). Concerning the rheological behavior, experimental observations indicated that the addition of fillers significantly altered the melt viscosity during the compounding process. Specifically, the incorporation of zeolite led to the most pronounced increase in viscosity. In contrast, the citric acid–crosslinked carboxymethyl cellulose matrix contributed to only a modest viscosity increase. As supported by the literature [57], this behavior is primarily attributed to the filler size effect; the highly dispersed, smaller sub-micron particles of zeolite impose a significantly greater steric hindrance on the long-range fluidic cooperative movement of the polymer melt compared to the micro-scale sheet- or flake-like structures of the C-En-CA matrix. As shown in Figure S2, raw PLA pellets were transparent, whereas Z-En/PLA and C-En-CA/PLA pellets appeared translucent due to the incorporation of Alcalase-immobilized composites. Notably, C-En-CA/PLA pellets exhibited yellow coloration, which intensified as the extrusion temperature increased. This discoloration is attributed to CMC, likely caused by accelerated partial thermal decomposition of CMC with reduced crystallinity induced by the freeze-drying process [58].

Cross-sectional morphologies of Z-En/PLA and C-En-CA/PLA prepared at 210 °C were examined. The cross-sectional scanning electron microscopy (SEM) image of Z-En/PLA (Figure 4b) exhibits relatively round domains, suggesting that the ceramic-based Z-En did not significantly increase melt viscosity during extrusion. Energy-dispersive X-ray spectroscopy (EDS) mapping (Figure 4c) confirms the presence of micron-sized Z-En particles dispersed within the PLA matrix, as evidenced by the distribution of Al elements (blue), serving as a zeolite-specific marker. The low-magnification EDS mapping image (Figure 4d) further demonstrates uniform dispersion throughout the matrix. In contrast, the cross-sectional image of C-En-CA/PLA shows increased pellet diameter with noticeable eccentricity (Figure 4e). EDS mapping of the Na element (purple), characteristic of CMC, reveals that C-En-CA forms sheet-like domains on the order of several tens of micrometers (Figure 4f). These larger grains are consistently observed in the low-magnification Na-element EDS mapping image. For comparison, Figure S3 presents the cross-section of a neat PLA pellet, which exhibits an oval geometry with a relatively smooth fracture surface.

Because of the harsh thermal conditions during the masterbatch fabrication process, verifying whether the immobilized enzyme retained its ability to accelerate PLA degradation was necessary. Although quantifying the retained enzymatic activity at each processing temperature would provide ideal insights, the exact quantification of active Alcalase embedded within the highly viscous polymer matrix is technically not feasible at this stage. Instead, following established methodologies in highly esteemed literature [36,59], enzyme survival was evaluated via indirect but definitive bio-catalytic/hydrolytic activity tests (i.e., depolymerization and biodegradation behavior).

**Figure 5:**
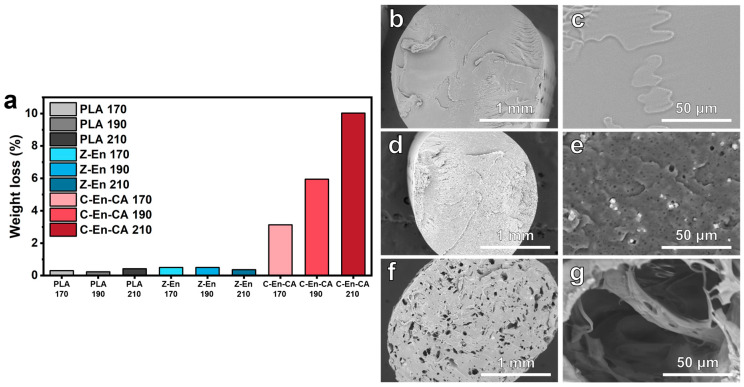
Evaluation of enzymatic degradation performance and morphological evolution of PLA masterbatches after 3 weeks of hydrolytic buffer degradation. (**a**) Quantitative weight loss of neat PLA, Z-En/PLA, and C-En-CA/PLA pellets after 3 weeks of incubation in a 50 °C buffer solution, showing significantly enhanced degradation for C-En-CA/PLA as processing temperature increases (up to 10.03% at 210 °C). (**b**–**g**) Cross-sectional SEM analysis after degradation (210 °C samples): (**b**,**c**) Neat PLA showing minimal surface changes, consistent with its negligible weight loss; (**d**,**e**) Z-En/PLA maintaining its overall shape but exhibiting localized submicron-to-micron-sized pores; (**f**,**g**) C-En-CA/PLA showing dramatic structural disintegration with large-scale pores (hundreds of micrometers), validating the high enzyme activity retention and accelerated degradation facilitated by the CMC entrapment system.

Figure 5a compares the weight loss after a degradation test conducted in buffer solution at 50 °C for 3 weeks. Neat PLA exhibited negligible weight loss (<0.5%) despite exposure to elevated temperature and aqueous buffer conditions. Z-En/PLA samples showed slightly increased weight loss at processing temperatures of 170 °C and 190 °C; however, the increments were limited to less than 0.3%. Moreover, the weight loss further decreased when processed at 210 °C. These results suggest that enzyme adsorption onto zeolite contributes only marginally to PLA degradation, particularly at processing temperatures approaching 210 °C. In contrast, incorporation of C-En-CA significantly enhanced PLA degradation. The weight loss of PLA increased progressively with processing temperature: 3.13% (170 °C), 5.95% (190 °C), and 10.03% (210 °C). As shown in Figure S4, all pellets became opaque after degradation. Notably, C-En-CA/PLA samples turned distinctly white, which is likely attributed to thermally degraded CMC components leaching out during the degradation process.

The synergistic protection afforded by the CMC–citric acid network involves both the tailored pre-treatment of the enzyme and the structural confinement provided by the crosslinked matrix, thereby addressing the specific thermal inactivation pathways. Specifically, the inhibition of moisture loss and conformational rigidity plays a critical role, as it is well-established that fully dehydrated enzymes exhibit extreme conformational rigidity, which drastically suppresses the prevalent covalent and non-covalent reactions responsible for irreversible thermal inactivation in aqueous solutions [45]. By employing lyophilization prior to the compounding process, the Alcalase was brought into a fully dehydrated state. Additionally, this strategy prevents protein unfolding and aggregation via nanocompartmentalization. The three-dimensionally crosslinked CMC–citric acid network physically encages and isolates individual enzyme molecules within its dense polymeric matrix. This spatial confinement restricts the molecular mobility of the enzyme, effectively hindering protein unfolding even when exposed to 210 °C. Furthermore, because the enzyme molecules are physically segregated within the rigid crosslinked cages, intermolecular collisions are blocked, successfully preventing irreversible protein aggregation. This protection is further enhanced by the short thermal exposure duration, as the industrial melt compounding process is highly transient and lasts only a few minutes, minimizing the kinetics of any potential thermal degradation. In summary, the dehydration-induced chemical resistance of the enzyme and the physical confinement of the crosslinked CMC–citric acid matrix synergistically protect the enzyme from unfolding and aggregation under the harsh thermal conditions of 210 °C.

Cross-sectional morphologies of neat PLA, Z-En/PLA, and C-En-CA/PLA prepared at 210 °C after 3 weeks of hydrolytic buffer degradation are shown in Figure 5b–g. Low- and high-magnification SEM images of neat PLA pellets (Figure 5b,c) revealed no discernible differences compared to undegraded samples, confirming minimal hydrolytic degradation. Z-En/PLA pellets maintained a similar overall cross-sectional shape under low magnification (Figure 5d), although submicron- to micron-sized pores were observed in high-magnification images (Figure 5e), indicating limited localized degradation. In contrast, C-En-CA/PLA pellets exhibited dramatic morphological changes, with pores on the order of hundreds of micrometers (Figure 5f,g). These observations are fully consistent with the quantitative weight-loss results.

Figure S5 presents mechanically fractured cross-sections of neat PLA, Z-En/PLA, and C-En-CA/PLA pellets at the same magnification as in Figure 5b–g. Neat PLA showed no evidence of degradation (Figure S5a,b). Z-En/PLA rarely exhibited small pores, likely due to limited buffer penetration and insufficient enzymatic activity of zeolite-adsorbed Alcalase (Figure S5c,d). In contrast, C-En-CA/PLA displayed morphology comparable to the degraded pellet cross-sections, reflecting progressive internal degradation. Although crosslinking was introduced by CA, the CMC host matrix remains hydrophilic and hygroscopic [60], enabling effective buffer infiltration and subsequent enzyme activation (Figure S5e,f). Therefore, entrapment within the CMC matrix is substantially more effective than adsorption onto zeolite for preserving enzyme activity during high-temperature processing while maintaining degradation functionality.

**Figure 6:**
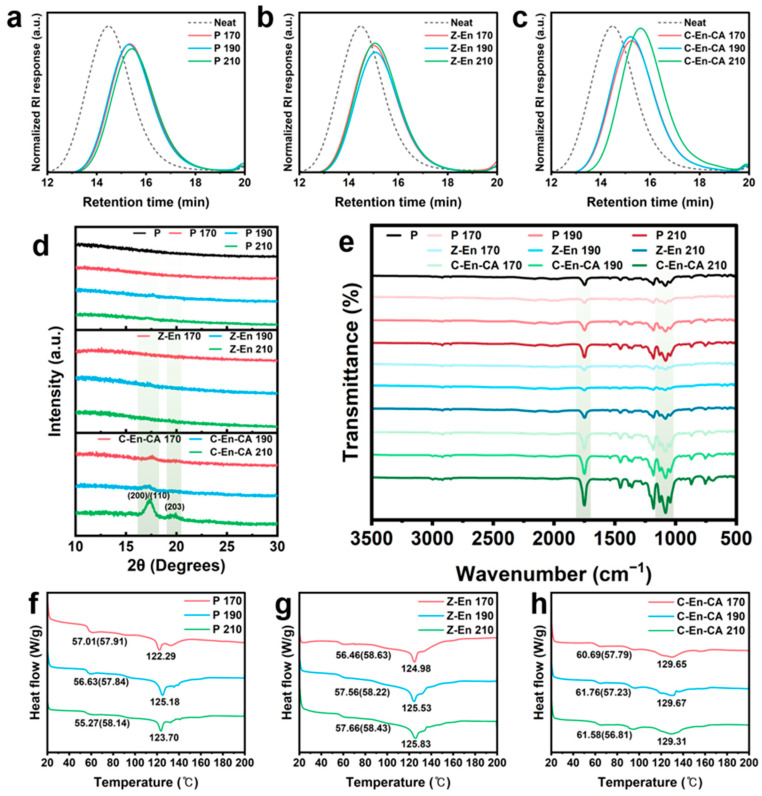
Molecular and structural evolution of PLA masterbatches under different processing temperatures. (**a**–**c**) GPC chromatograms: Normalized RI response curves for (**a**) neat PLA, (**b**) Z-En/PLA, and (**c**) C-En-CA/PLA, showing a significant shift toward higher retention times (molecular weight reduction) primarily due to thermomechanical chain scission during melt extrusion. (**d**) Crystallographic analysis: WAXD patterns showing predominantly amorphous structures for neat PLA and Z-En/PLA, whereas C-En-CA/PLA exhibits distinct α-crystal diffraction peaks at 2θ ≈ 16.5° and 18.5°, indicating degradation-induced recrystallization. (**e**) Molecular structural changes: ATR-FT-IR spectra highlighting the transmittance decrease of carbonyl (C=O) and C–O–C stretching bands with increasing temperature, indicative of ester bond cleavage and new end-group formation. (**f**–**h**) Thermal properties: DSC thermograms for (**f**) neat PLA, (**g**) Z-En/PLA, and (**h**) C-En-CA/PLA; the broadened endotherms and higher T_g_ and T_m_ values in (**h**) reflect the formation of heterogeneous crystalline lamellae, consistent with the WAXD and molecular weight analysis results.

Molecular and structural characterizations were conducted to evaluate chain scission and recrystallization induced by extrusion and the subsequent degradation test. Molecular weights were measured by gel permeation chromatography (GPC). As shown in Figure 6a–c and Table S2, the GPC curves of neat PLA, Z-En/PLA, and C-En-CA/PLA masterbatches were shifted toward higher retention times (from 14.4 min to >15 min), indicating molecular weight reduction compared to as-received PLA (Mn = 151,150 g mol^−1^). After extrusion, the molecular weights decreased to approximately 50,024–83,941 g mol^−1^, corresponding to nearly a 50% reduction. From the GPC trends alone, it was difficult to clearly distinguish the enzymatic contribution to degradation. Moreover, the degradation-tested neat PLA and Z-En/PLA samples did not exhibit noticeable morphological differences in their fractured cross-sections (Figure S5). Therefore, the observed molecular weight reduction is primarily attributed to thermomechanical chain scission caused by high-shear melt compounding during extrusion [61]. However, the C-En-CA/PLA masterbatch processed at 210 °C exhibited the lowest molecular weight among all tested groups, hinting at an additional degradation pathway. This behavior can be interpreted through a “heterogeneous contact degradation” mechanism; the entrapped Alcalase operates predominantly via direct localized contact with the adjacent polyester chains within its immediate microenvironment. Consequently, while the polymer chains in direct contact with the enzyme undergo intense enzymatic cleavage, the uncontacted bulk regions remain relatively unaffected, which leaves the overall average molecular weight of the macro-bulk sample largely unchanged in the GPC profile.

The crystallographic structures were examined using wide-angle X-ray diffraction (WAXD). As shown in Figure 6d, the as-received PLA, extruded neat PLA, and Z-En/PLA samples exhibited broad halos without distinct diffraction peaks, indicating predominantly amorphous structures. In contrast, C-En-CA/PLA masterbatches displayed characteristic diffraction peaks, corresponding to the (200)/(110) and (203) planes of PLA α-crystals, [62,63,64]. These peaks shifted from the typical 2θ positions of approximately 16.5° and 19.0° to higher values of 17.3° and 19.7°, respectively. This high-angle shift suggests a tighter yet slightly distorted crystalline packing induced by the rearrangement of shorter, enzymatically cleaved chains. These peaks were most pronounced in samples fabricated at 210 °C. The enhanced crystallinity suggests degradation-induced crystallization, enabled by reduced molecular weight and increased chain mobility, which facilitates crystalline organization in the polyester matrix [65].

Molecular structural changes were further evaluated using Fourier-transform infrared (FT-IR) spectroscopy. Before proceeding with the analysis, it should be noted that the chemical confirmation of the CMC–citric acid crosslinking, specifically the carbonyl stretching band (C=O) originating from the crosslinking typically appears near ~1750 cm^−1^ [66]. However, because the PLA matrix constitutes the vast majority of the blend (90 wt%), the extremely dominant and intense ester carbonyl stretching peaks from the PLA backbone [67] completely overlap with and mask the signals from the embedded CMC matrix. Consequently, direct tracking of the crosslinking density changes via FT-IR within the composite was technically prohibited due to this matrix shielding effect. Nevertheless, the successful crosslinking of the CMC–citric acid hydrogel framework itself was pre-validated prior to compounding based on well-established protocols in the literature [66], and its operational success within the masterbatch is indirectly yet definitively evidenced by the superior thermal protection of the enzyme at 210 °C. The C=O (~1750 cm^−1^) and C–O–C stretching region (1000–1300 cm^−1^), normalized to the CH_3_ bending peak at 1455 cm^−1^, are associated with the formation of new carboxylic acid and ketone end groups resulting from ester bond cleavage in the PLA backbone, as well as structural alterations due to chain scission [68,69]. As shown in Figure 6e, the transmittance of these bands greatly decreased with increasing processing temperature. The enhanced C=O and C–O–C signals can be interpreted as a synergistic effect of thermomechanical degradation during extrusion and limited abiotic hydrolysis during the degradation test.

Differential scanning calorimetry (DSC) was performed under a nitrogen atmosphere to evaluate thermally induced structural changes. The reported glass transition temperature (T_g_) and melting temperature (T_m_) of pure PLA are 59–61 °C and 154–>160 °C, respectively, depending on crystallinity [70,71,72]. As shown in Figure 6f,g, neat PLA and Z-En/PLA masterbatches exhibited T_g_ values of 55–58 °C and T_m_ values of 122–126 °C, consistent with their low crystallinity. In contrast, C-En-CA/PLA masterbatches displayed a higher Tg (~61 °C) and higher Tm (~130 °C), along with broadened and shallow glass transition and melting endotherms. This behavior indicates the formation of heterogeneous crystalline structures with a broad distribution of lamellar thicknesses, consistent with degradation-induced recrystallization [73]. These findings are in good agreement with the WAXD results (Figure 6d). Based on the combined morphological, crystallographic, and molecular structural analyses, C-En-CA/PLA subjected to degradation exhibited not only macroscopic shape changes but also significant microstructural modification. In contrast, neat PLA and Z-En/PLA showed minimal structural evolution, confirming that CMC-based entrapment effectively preserves enzymatic functionality while enabling post-processing degradation behavior.

Enzyme-immobilized PLA masterbatches prepared at 210 °C were further blended with PBAT to demonstrate a practical application of the immobilized enzyme system. PBAT/PLA blends have been extensively investigated owing to their inherent biodegradability and minimal adverse effects on soil during degradation [74]. Moreover, PBAT exhibits higher degradation potential than PLA due to its flexible aliphatic–aromatic chain structure and greater susceptibility to hydrolysis by serine hydrolases [75,76]. PBAT blended with PLA masterbatches was processed into 300 μm-thick films using a T-die extruder (Figure S6). The concentrations of enzyme-immobilized masterbatches (Z-En and C-En-CA) were 1 and 2 wt%. Despite the yellowish coloration of the C-En-CA/PLA masterbatch, the PBAT blended with C-En-CA/PLA exhibited no noticeable color difference compared to neat PBAT, maintaining its white appearance.

**Figure 7:**
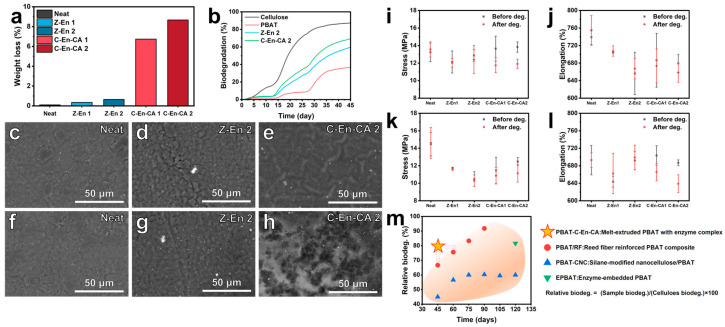
Evaluation of enzymatic degradation, mechanical performance, and comparative biodegradability of PBAT-based composite films after 15 days in a 50 °C buffer solution and under industrial composting conditions (58 °C) over 45 days. (**a**) Gravimetric weight loss after 15 days in a 50 °C buffer, highlighting the superior degradation of the C-En-CA series (up to 8.67% at 2 wt%) compared to the negligible loss in the neat and Z-En series. (**b**) Aerobic biodegradation profiles under industrial composting conditions (58 °C) over 45 days; the C-En-CA system achieved 69.4% absolute biodegradation, significantly outperforming neat PBAT (36.9%) and the Z-En system. (**c**–**h**) Surface morphological evolution: SEM images of (**c**–**e**) as-prepared films showing smooth surfaces and (**f**–**h**) after 15-day degradation, where the C-En-CA system (**h**) displays distinct porous structures consistent with active enzymatic erosion. (**i**–**l**) Mechanical property changes before and after. 15-day degradation: Tensile stress and elongation at break measured in (**i**,**j**) machine direction (MD) and (**k**,**l**) transverse direction (TD), revealing significant strength and ductility reduction specifically for the C-En-CA series due to degradation-induced defects. (**m**) Comparative analysis of relative biodegradability: Comparison with reported PBAT-based systems (RF, CNC, and EPBAT), demonstrating that the C-En-CA system achieves high relative biodegradation (79.5%) in a shorter timeframe (45 days), matching the efficiency of long-term enzyme-embedded systems [80,81,82].

The degradation behavior of PBAT/PLA blended films was evaluated after 15 days in a 50 °C buffer solution (Figure 7a). The neat PLA and Z-En series showed less than 1% weight loss across all compositions, indicating limited degradation. In contrast, the C-En-CA series demonstrated pronounced degradation, with 1 wt% and 2 wt% samples showing weight losses of 6.74% and 8.67%, respectively. Notably, the effective enzyme content was extremely low, below 0.2 wt% even for the 2 wt% C-En-CA sample, considering that the enzyme-immobilized zeolite or CMC content in the PLA masterbatch was only 10 wt%. Despite this minimal loading, the immobilized Alcalase significantly accelerated polymer degradation, highlighting the efficiency of the entrapment strategy. Furthermore, because the masterbatch incorporation in the final film was highly diluted (1 to 2 wt%), the actual content of the hydrophilic CMC carrier in the final film was only 0.1 to 0.2 wt%. Consequently, any physical dissolution or leaching of the CMC matrix into the buffer was strictly limited and could account for at most 0.1 to 0.2 wt% of the total film mass. Therefore, the substantial weight loss observed exclusively in the C-En-CA series directly represents active, enzyme-catalyzed ester bond cleavage within the polymer matrix, rather than simple carrier leaching or abiotic hydrolysis.

The ultimate aerobic biodegradability of T-die films containing 0 and 2 wt% enzyme-immobilized PLA masterbatches was further evaluated under simulated industrial composting conditions. Industrial composting was selected over home composting for two primary reasons: (i) industrial composting conditions (58 °C) provide the necessary thermodynamic energy to accelerate the abiotic hydrolysis of semi-crystalline polyesters like PLA and PBAT, which is practically stagnant under mesophilic home composting temperatures (20–30 °C), and (ii) testing under these high-temperature conditions is strictly required to comply with international biodegradability standards such as EN 13432, making it the most commercially relevant end-of-life scenario for our masterbatches. Additionally, industrial facilities utilize gas treatment systems to mitigate uncontrolled greenhouse gas emissions, which remain a major environmental concern in unmanaged home composting systems [77,78].

Film specimens were buried in a standardized compost matrix maintained at 58 °C under controlled moisture and aeration to simulate the thermophilic phase of industrial composting, in strict accordance with the KS M ISO 14855-1 standard protocol. This elevated temperature of 58 °C is specified to maximize the metabolic activity and population growth of thermophilic microorganisms (such as Bacillus and Actinobacteria), while simultaneously increasing the chain mobility of the polyester matrix to facilitate enzymatic cleavage [11]. Detailed information on the biodegradation degree of the PBAT-based films under industrial composting conditions is provided in Table S3. The absolute biodegradation profiles during 45 days under industrial composting conditions (Figure 7b) revealed slightly different trends compared to the buffer degradation test. Both Z-En and C-En-CA systems showed comparable activity up to day 13; however, beyond this point, C-En-CA exhibited superior degradation performance. Around the 30th day, which represents a critical intermediate stage, the second inflection and subsequent acceleration in decomposition were observed for the neat PBAT, Z-En, and C-En-CA samples. On day 30, neat PBAT showed limited relative biodegradation compared to cellulose (22.6%), whereas 2 wt% Z-En and C-En-CA samples achieved 47.1% and 58.9% of relative biodegradation, respectively. It is noteworthy that the biodegradation curves of cellulose and PBAT-based samples exhibit typical decomposition behaviors with one inflection point for cellulose and two for the PBAT matrix, as documented in the literature [79]. Notably, the C-En-CA composite exhibited a significantly boosted degradation rate after this 30-day intermediate point owing to the successful and stable immobilization of the enzyme, demonstrating its superior long-term performance up to the final 45-day mark. After 45 days, neat PBAT reached 42.3% relative biodegradation, while the 2 wt% C-En-CA sample achieved 79.5% relative biodegradation, which corresponds directly to an absolute biodegradation value of 69.4%. This outstanding performance is particularly significant because it demonstrates that the immobilized enzyme successfully retained substantial biocatalytic activity even after undergoing two sequential high-temperature extrusion steps. Ultimately, this substantial acceleration beyond the baseline polymer controls confirms that the surviving, active enzyme plays the dominant role in initiating and accelerating the chemical degradation of the polyester matrix.

Surface morphologies of films before and after degradation (buffer-tested samples to avoid soil artifacts) are shown in Figure 7c–h. As-prepared neat PBAT and PBAT/PLA composite films exhibited smooth, defect-free surfaces (Figure 7c–e). After degradation, neat PBAT and 2 wt% Z-En/PLA composite films maintained similar surface morphologies. In contrast, C-En-CA/PLA composite films displayed significantly porous surfaces after degradation (Figure 7f–h), consistent with the substantial weight loss observed in Figure 7a.

Mechanical performance was evaluated using a universal testing machine along both the machine direction (MD) and transverse direction (TD). The specimens were prepared in a dogbone shape as illustrated in Figure S7 before testing. In the MD (Figure 7i,j), the tensile strengths of neat PBAT and Z-En/PLA composite films remained essentially unchanged after degradation. However, C-En-CA/PLA composite films exhibited a significant tensile strength reduction of approximately 18%. The elongation at break of neat PBAT and Z-En/PLA composite films slightly increased after degradation, possibly due to minor plasticization effects. In contrast, the elongation of C-En-CA/PLA composite films decreased, likely due to degradation-induced pore formation and structural defects. A similar trend was observed in the TD (Figure 7k,l). Tensile strengths of neat PBAT and Z-En/PLA composites remained stable, whereas C-En-CA/PLA composites showed noticeable strength reduction. Likewise, breaking strains increased slightly for neat PBAT and Z-En/PLA composites but decreased for C-En-CA/PLA composites.

The relative biodegradation of the C-En-CA system was compared to other reported PBAT-based composites to further contextualize these results (Figure 7m). All compared studies were conducted under similar aerobic composting conditions at 58 °C. While most evaluations, including the present work, utilized standard cellulose as a reference, the PBAT-CNC system employed microcrystalline cellulose (MCC) as its control [80]. Despite these standardized testing environments, typical filler-based composites, such as PBAT-CNC and PBAT/RF, exhibited either a plateau effect or significantly delayed biodegradation [81]. By contrast, the C-En-CA system achieved 79.5% (denoted as ⛤) relative biodegradation within only 45 days. Notably, this substantial degradation was triggered by a loading of only 0.20 wt% of the enzyme complex within the T-die films. This outcome is significant as it matches the ultimate degradation efficiency of enzyme-embedded systems (EPBAT) [82], which required a much longer period of 120 days to reach comparable levels. Integrating enzyme complexes during melt extrusion effectively shortens the induction period and boosts degradation, providing a practical route for time-efficient industrial composting.

Theoretically, our C-En-CA platform can be extended to other biodegradable polyesters such as poly(butylene succinate) (PBS), poly(caprolactone) (PCL), or polyhydroxyalkanoates (PHAs). Since the masterbatch is compounded via melt blending, this stabilization strategy can be adapted to other biodegradable polymers by adjusting the compounding temperature to match the melting point of the respective carrier matrix (such as PCL with a lower melting temperature, or PBS and PHAs). Furthermore, our masterbatch-based process is fundamentally designed to be compatible with existing polymer processing infrastructures. Because the enzyme-loaded CMC is formulated into standard compounding pellets, these pellets can be introduced directly into industrial-scale film extrusion, fiber spinning, or injection-molding hoppers just like conventional functional masterbatches. While executing fiber spinning or injection-molding trials on an industrial scale is beyond the scope of this laboratory-level study, the use of standard melt-compounding and pelletization suggests high compatibility and technical viability for these diverse manufacturing operations in the future.

## 3. Conclusions

This study establishes a practically viable, masterbatch-based biocatalytic platform for programmable self-degradation of bio-based polyesters under industrially relevant processing conditions. Moving beyond simple thermal stabilization, we demonstrate true process survivability: the C-En entrapment architecture withstands high-shear twin-screw extrusion at temperatures up to 210 °C while retaining catalytic functionality. Hydrolytic degradation, supported by GPC-verified chain scission, produced a 10.03% mass loss in 0.05 M Tris-HCl buffer (pH 8.0) after 3 weeks, confirming post-extrusion enzyme activity and bulk matrix deterioration. Cross-sectional SEM observations suggest that the hydrophilic CMC matrix facilitates swelling-induced aqueous diffusion pathways, increasing the effective interfacial reaction area, while XRD analysis revealed degradation-induced crystallization resulting from enhanced chain mobility after enzymatic scission. Successful translation into PBAT T-die films, achieving 79.5% biodegradation within 45 days under industrial composting conditions, underscores the scalability and manufacturing compatibility of this approach. Nonetheless, we acknowledge that these accelerated degradation profiles are specifically relevant to controlled, thermophilic industrial composting environments (58 °C). Extrapolating these results to natural environments, such as marine, freshwater, or soil ecosystems, requires further investigation, which remains a key focus of our ongoing and future research. Collectively, this work defines a robust design framework for smart, self-degradable polymer systems aligned with circular materials engineering.

## 4. Experimental Section

### 4.1. Materials and Enzyme Immobilization

PLA (LX930) was purchased from Total Corbion (Gorinchem, The Netherlands) and used as the biodegradable matrix for the masterbatch preparation. PBAT was supplied by SK leaveo (Seoul, Republic of Korea). Zeolite (No. 96096) and citric acid (CA) were obtained from Sigma-Aldrich (St. Louis, MO, USA). CMC (7M31F) was provided by Ashland (Wilmington, DE, USA). Alcalase 2.5L, a commercial liquid enzyme formulation of a serine endo-type protease (subtilisin) derived from Bacillus licheniformis, was supplied by Novozymes (Bagsværd, Denmark) and used as the model protease. Tris–HCl buffer (1 M, pH 8.0), purchased from Bioneer (Daejeon, Republic of Korea), was diluted with deionized water prior to use. All reagents were used as received unless otherwise specified.

Alcalase, zeolite, and buffer were mixed at a weight ratio of 1:1:20 and stirred at 1000 rpm for 12 h. The suspension was filtered under reduced pressure and dried under vacuum to obtain Z-En. A 2 wt% aqueous CMC solution was prepared, and Alcalase was added at a 1:1 weight ratio relative to CMC. CA was introduced as a crosslinking agent. The mixture was stirred, freeze-dried, pulverized, and sieved through a 150 μm mesh.

Immobilization efficiency was determined using a Pierce™ BCA Protein Assay Kit (Thermo Fisher Scientific, Waltham, MA, USA). A calibration curve was established using The assay is based on the reduction of Cu^2+^ by proteins, followed by formation of a purple chelate complex with bicinchoninic acid (BCA) [83]. A calibration curve was established using bovine serum albumin (BSA) (0–2000 µg mL^−1^, R^2^ > 0.99). Absorbance was measured at 562 nm using a SpectraMax iD3 microplate reader (Molecular Devices, San Jose, CA, USA). Immobilization efficiency was calculated as

$$The\;immobilization\;efficiency\left(\%\right)=\frac{C_{0}-C_{s}}{C_{0}}\times 100$$

where $C_{0}$ is the initial enzyme added during the immobilization process, and $C_{s}$ is the residual concentration in supernatant [84].

Surface chemical states were analyzed by XPS (Quantum 2000, ULVAC-PHI, Chigasaki, Japan) using monochromatic Al Kα radiation (1486.6 eV, 15 kV, 25 W) with a beam size of 100 μm. Thermal stability was evaluated via TGA (Q500, TA Instruments, New Castle, DE, USA) under N2 from room temperature to 800 °C at a ramping rate of 10 °C min^−1^, and analyzed using TA Universal Analysis software (TA Instruments, New Castle, DE, USA). An isothermal test at 200 °C for 60 min, following rapid heating at 100 °C min^−1^, was performed to simulate melt-processing conditions.

### 4.2. Masterbatch and Film Fabrication

Masterbatches were prepared using a twin-screw extruder (BA-11, BauTech, Uiwang, Republic of Korea). PLA was dry-mixed with 10 wt% Z-En or C-En-CA and processed at 170, 190, or 210 °C. Samples were designated as P 170–P 210 (neat PLA), Z-En 170–Z-En 210, and C-En-CA 170–C-En-CA 210.

PBAT films were fabricated using a twin-screw extruder equipped with a T-die attachment (STS32HS-40-2V-SF, Hankook EM, Incheon, Republic of Korea). Masterbatch contents were 1 and 2 wt%, denoted as Z-En 1–2 and C-En-CA 1–2.

### 4.3. Degradation Tests

For hydrolytic degradation, samples were immersed in 0.05 M Tris–HCl buffer (pH 8.0) at a liquid-to-solid ratio of 20:1 and incubated at 50 °C for 3 weeks (pellets) or 15 days (films). Specimens were washed and dried to constant weight at 80 °C.

Aerobic biodegradability of the films was evaluated under composting conditions at 58 °C with controlled moisture and aeration. Microcrystalline cellulose served as the reference. CO_2_ evolution was monitored, and biodegradation percentages were calculated relative to theoretical CO_2_ production. To directly evaluate the comparative degradation performance against the standard reference, the relative biodegradation was further calculated according to the following equation:

$$\mathrm{Relative}\;\mathrm{biodegradation}=\frac{\mathrm{Sample}\;\mathrm{biodegradation}}{\mathrm{Cellulose}\;\mathrm{biodegradation}}\times 100$$

where Sample biodegradation and Cellulose biodegradation denote the absolute carbon conversion percentages of the polymer film sample and the microcrystalline cellulose reference, respectively.

### 4.4. Characterization

Morphology was examined using SEM (TM4000Plus, Hitachi, Tokyo, Japan) at 15 kV in backscattered electron (BSE) mode. Elemental mapping was performed using EDS (Oxford Instruments, Abingdon, UK) analyzed with AZtec software (Oxford Instruments, Abingdon, UK). Molecular weights were measured by GPC (Waters 1525, Waters Corporation, Milford, MA, USA) using THF at 0.3 mL min^−1^ and the data were analyzed using Empower software (Waters Corporation, Milford, MA, USA) calibrated with polystyrene standards. WAXD patterns were collected using a Rigaku SmartLab (Rigaku Corporation, Tokyo, Japan) (Cu Kα, 5–80° 2θ) and processed using SmartLab Studio II software (Rigaku Corporation, Tokyo, Japan). FT-IR spectra were obtained using a Nicolet iS 50 (Thermo Fisher Scientific, Waltham, MA, USA) and analyzed via OMNIC software (Thermo Fisher Scientific, Waltham, MA, USA). Thermal transitions were analyzed using a thermogravimetric analyzer (TGA; Q500, TA Instruments, New Castle, DE, USA) and DSC (Q100, TA Instruments, New Castle, DE, USA) operated with TA Universal Analysis software. Tensile properties were measured using an Instron 3343 universal testing machine (Instron, Norwood, MA, USA) operated via Bluehill software (Instron, Norwood, MA, USA) at a crosshead speed of 200 mm min^−1^ with at least three specimens per sample.

## Data Availability

The original contributions presented in this study are included in the article/Supplementary Material. Further inquiries can be directed to the corresponding authors.

## Supplementary Materials

The following supporting information can be downloaded at: https://www.mdpi.com/article/10.3390/polym18151830/s1, Figure S1. Morphologies and corresponding EDS Ca elemental mapping images of the immobilized enzyme systems: (a) SEM micrograph and (b) Ca elemental map of the Alcalase-loaded zeolite (Z-En) particles; (c) SEM micrograph and (d) Ca elemental map of the citric acid–crosslinked carboxymethyl cellulose (C-En-CA) matrix. The uniform distribution of the purple dots (representing Ca atoms derived from the enzyme structure) confirms the homogenous loading of Alcalase across and within both host scaffolds; Figure S2. Optical images of PLA, Z-En/PLA, and C-En-CA/PLA pellets fabricated via melt extrusion at various processing temperatures; Figure S3. Cross-sectional SEM images of a neat PLA pellet. (a) Low-magnification image showing the oval geometry and (b) high-magnification image of the relatively smooth fracture surface; Figure S4. Visual appearance of PLA and enzyme-integrated masterbatch pellets processed at various temperatures after 3 weeks of degradation in buffer solution at 50 °C; Figure S5. SEM micrographs of mechanically fractured cross-sections of neat PLA, Z-En/PLA, and C-En-CA/PLA pellets. (a,b) Neat PLA, (c,d) Z-En/PLA, and (e,f) C-En-CA/PLA; Figure S6. Optical images of neat PBAT and enzyme-integrated PBAT films fabricated via T-die extrusion; Figure S7. Dimensions and visual appearance of the dogbone-shaped specimen used for mechanical property measurements; Table S1. Surface atomic compositions of Z, Z-En, C, and C-En-CA determined by XPS; Table S2. Molecular weight properties of neat PLA and enzyme-integrated masterbatch pellets processed at various melt temperatures; Table S3. Biodegradation degree of PBAT-based films under industrial composting conditions.
