# Supplementary material for: Process-Compatible Immobilization of Thermolabile Enzymes for Self-Degradable Polyesters via Industrial Melt Compounding Conditions
Source: Polymers (Basel). 2026 Jul 26;18(15):1830. doi: 10.3390/polym18151830 (PMC13468501; doi:10.3390/polym18151830)
Supplement: Supplementary file 1 [file polymers-18-01830-s001.zip › polymers-4422969-supplementary.pdf]

Supporting Information to

Process-Compatible Immobilization of Thermolabile Enzymes for Self-Degradable Polyesters via Industrial Melt Compounding Conditions

Jeong Je Seo[1,2,†], In-Woo Nam[1,†], HyoJung Kim[1,2], Hajoong Kim[2], Jung Nam Im[1], Hyun Ju Oh[1], Jungyeon Kim[1], Byoung-Sun Lee[2,*,] Yeong Og Choi[1,*]

[1]*Textile Innovation R&D Department, Korea Institute of Industrial Technology, Ansan, 15588, Republic of Korea*

[2]*Department of Fiber System Engineering, Dankook University, Yongin 16890, Republic of Korea*

[†] These authors contributed equally to this work.

Table S1. Surface atomic compositions of Z, Z-En, C, and C-En-CA determined by XPS.

Sample	C1s (%)	N1s (%)	O1s (%)	Na1s (%)	Al2p (%)	Si2p (%)
Z	17.53	0.40	48.37	13.44	9.24	11.02
Z-En	28.04	2.94	42.68	8.51	8.34	9.49
C	58.56	0.02	34.57	6.62	0.22	0.00
C-En-CA	63.76	3.11	28.86	4.18	0.00	0.09

Table S2. Molecular weight properties of neat PLA and enzyme-integrated masterbatch pellets processed at various melt temperatures.

Sample	Mn	Mw	Mp	Mw/Mn
**Neat PLA**	80,803	151,150	124,462	1.87
**PLA 170**	35,907	62,724	55,846	1.74
**PLA 190**	37,593	65,167	57,594	1.73
**PLA 210**	33,437	58,879	52,448	1.76
**Z-En/PLA 170**	48,063	83,941	73,860	1.74
**Z-En/PLA 190**	44,052	78,866	69,621	1.79
**Z-En/PLA 210**	45,832	80,312	70,606	1.76
**C-En-CA/PLA 170**	37,838	69,338	60,475	1.83
**C-En-CA/PLA 190**	38,083	70,854	62,499	1.86
**C-En-CA/PLA 210**	27,447	50,024	44,591	1.82

Table S3. Biodegradation degree of PBAT-based films under industrial composting conditions.

	Sample	Biodegradation of standard cellulose, B_{std} (%)[1]	Biodegradation of sample, B_s (%)[2]	Relative biodegradation, B_{rel} (%)[3]
15 days	**Neat**		2.1	9.1
	**Z-En 2**	23.1	7.2	31.2
	**C-En 2**		10.6	45.9
30 days	**Neat**		18.2	22.6
	**Z-En 2**	80.5	37.9	47.1
	**C-En 2**		47.4	58.9
45 days	**Neat**		36.9	42.3
	**Z-En 2**	87.3	59.8	68.5
	**C-En 2**		69.4	79.5

[1]. Cellulose biodeg. (%): Biodegradation degree of the standard cellulose at each test time

[2]. Sample biodeg. (%): Average biodegradation degree of the PBAT-based film samples

[3]. Relative biodeg. (%): Relative biodeg.

$$\text{Relative biodeg.} = \frac{\text{Sample biodeg.}}{\text{Celluloes biodeg.}} \times 100$$

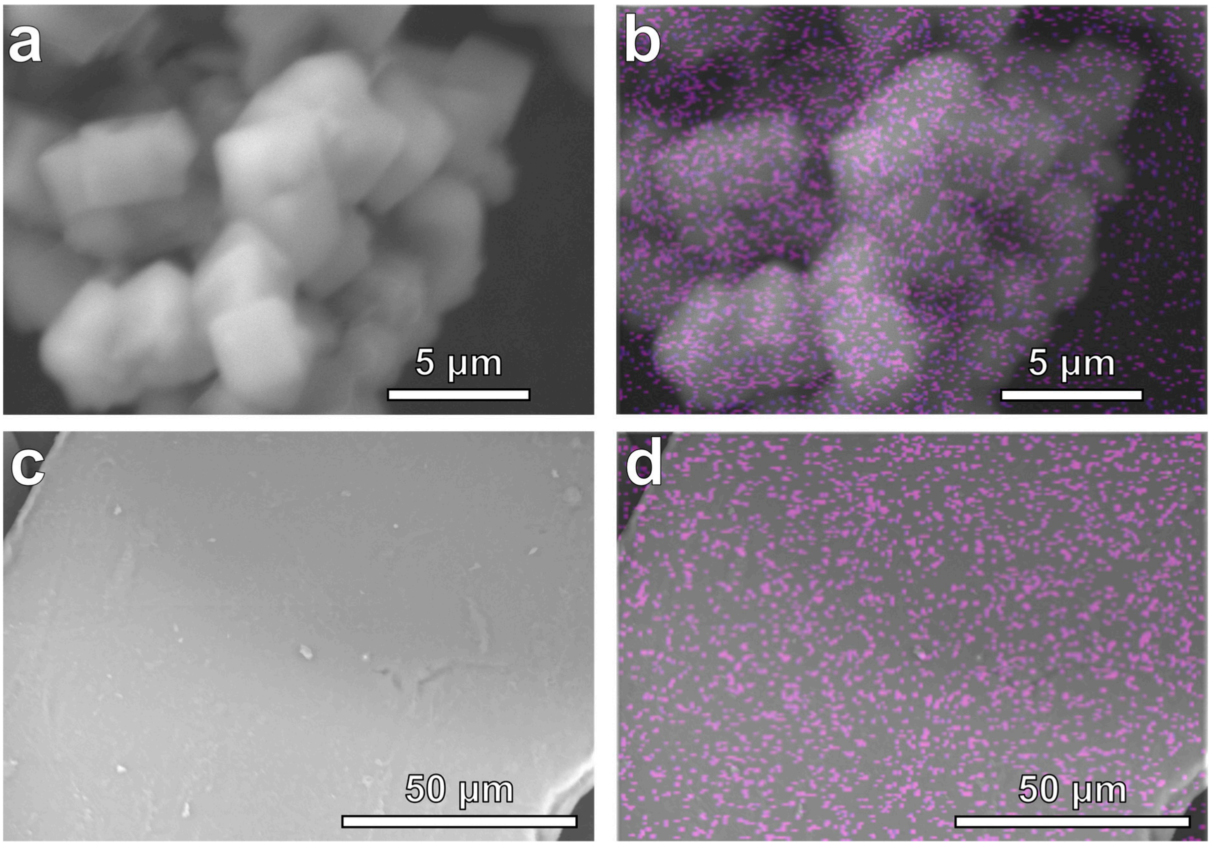

Figure S1. Morphologies and corresponding EDS Ca elemental mapping images of the immobilized enzyme systems: (a) SEM micrograph and (b) Ca elemental map of the Alcalase-loaded zeolite (Z-En) particles; (c) SEM micrograph and (d) Ca elemental map of the citric acid–crosslinked carboxymethyl cellulose (C-En-CA) matrix. The uniform distribution of the purple dots (representing Ca atoms derived from the enzyme structure) confirms the homogenous loading of Alcalase across and within both host scaffolds.

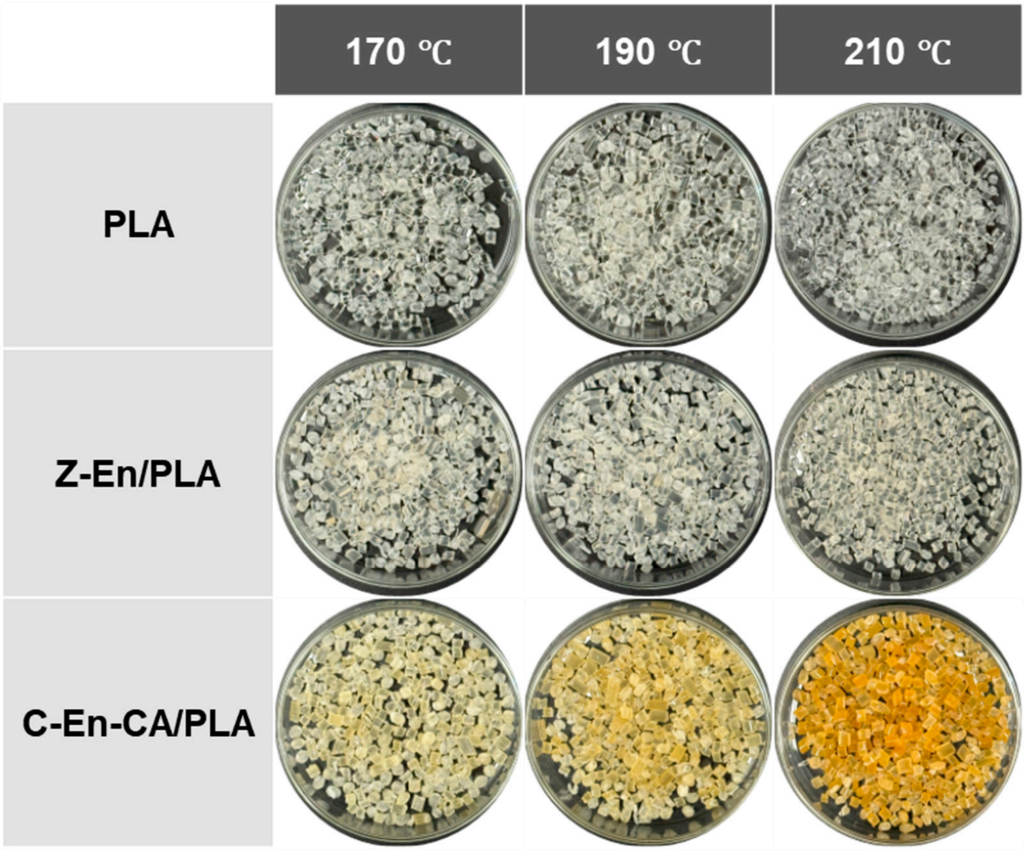

Figure S2. Optical images of PLA, Z-En/PLA, and C-En-CA/PLA pellets fabricated via melt extrusion at various processing temperatures.

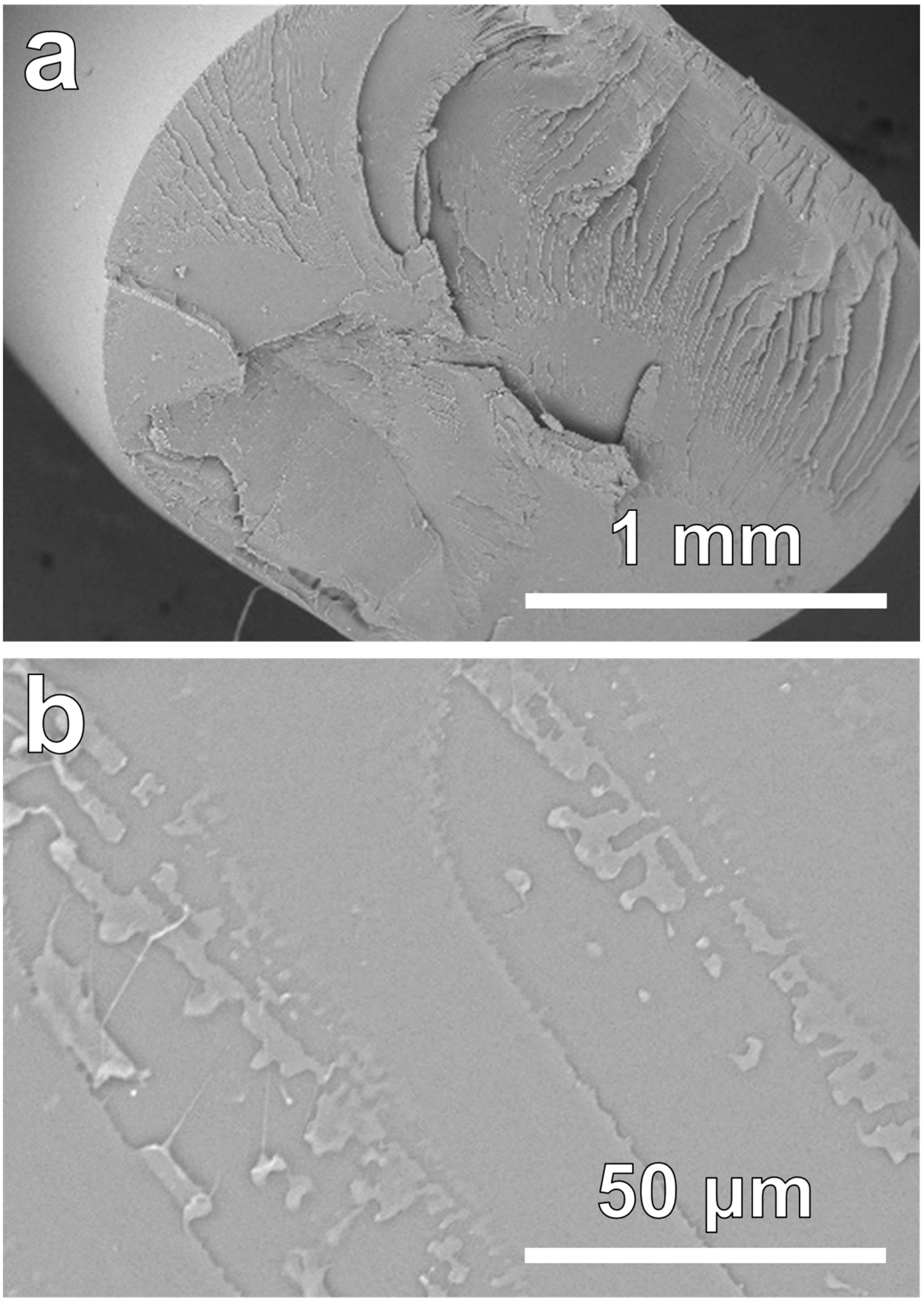

Figure S3. Cross-sectional SEM images of a neat PLA pellet. (a) Low-magnification image showing the oval geometry and (b) high-magnification image of the relatively smooth fracture surface.

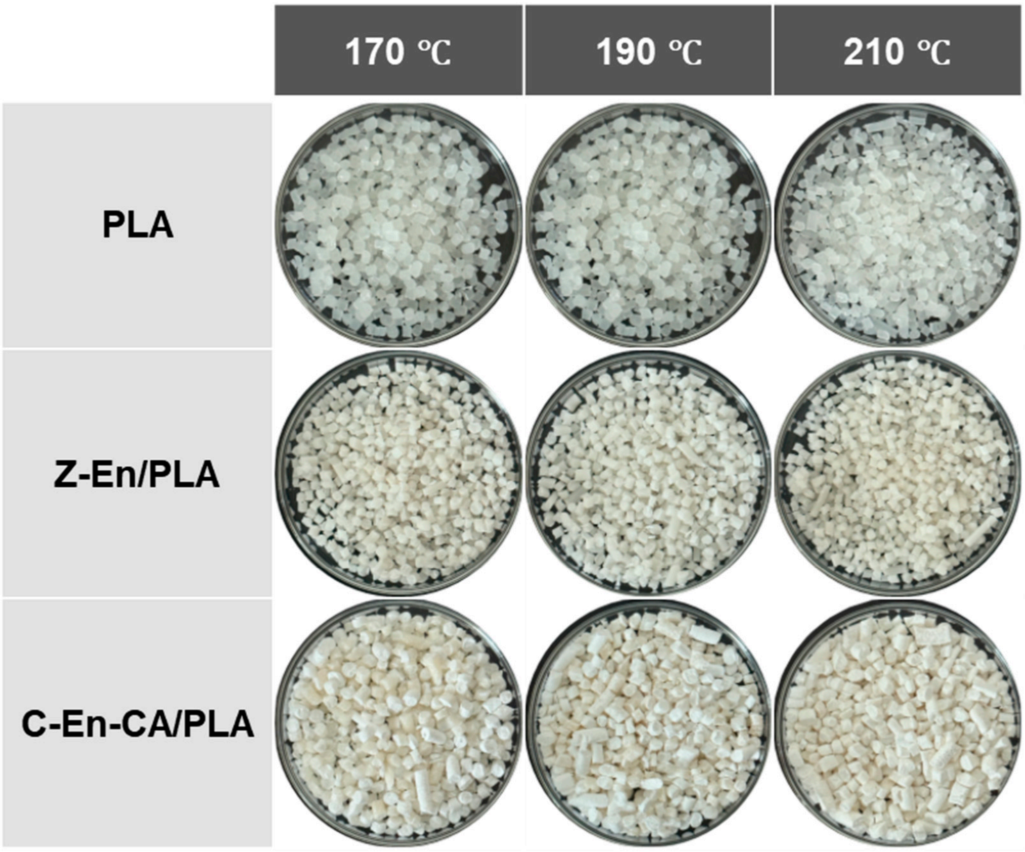

Figure S4. Visual appearance of PLA and enzyme-integrated masterbatch pellets processed at various temperatures after 3 weeks of degradation in buffer solution at 50 °C.

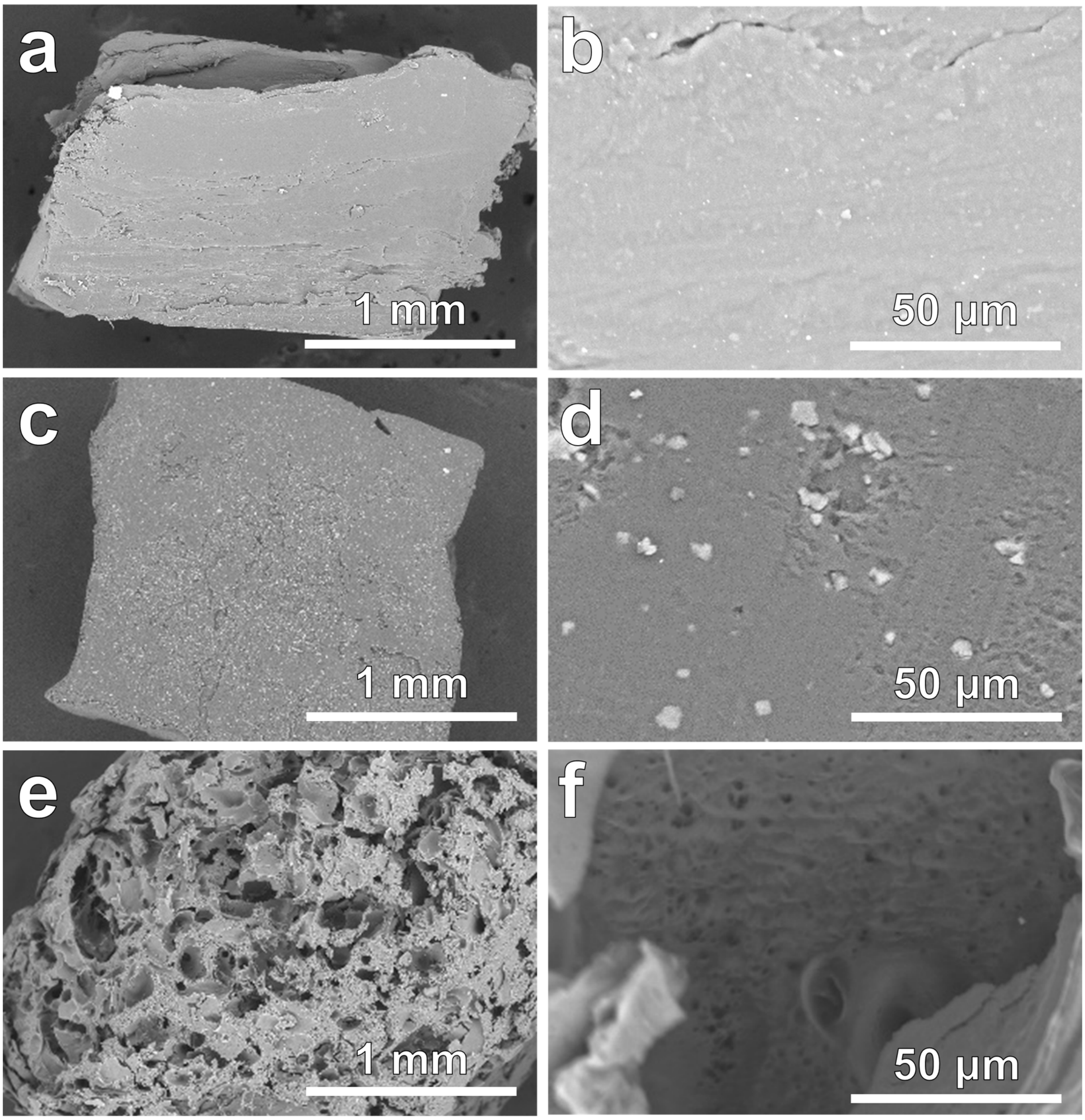

Figure S5. SEM micrographs of mechanically fractured cross-sections of neat PLA, Z-En/PLA, and C-En-CA/PLA pellets. (a, b) Neat PLA, (c, d) Z-En/PLA, and (e, f) C-En-CA/PLA.

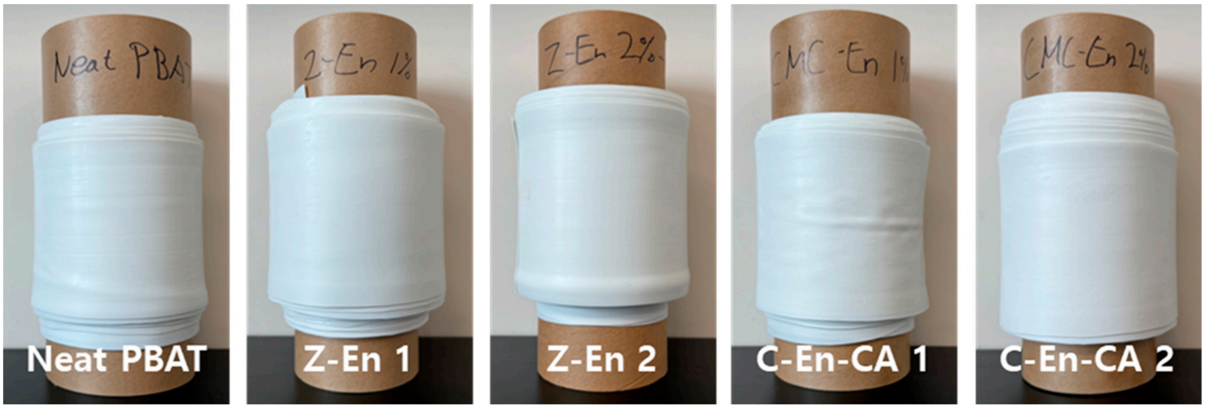

Figure S6. Optical images of neat PBAT and enzyme-integrated PBAT films fabricated via T-die extrusion.

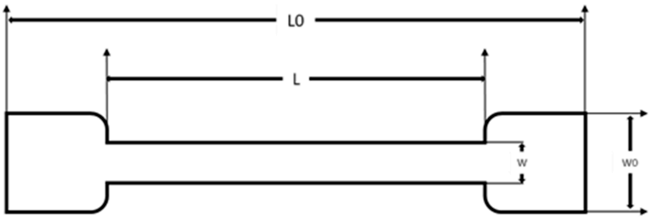

L	42 mm
L0	63 mm
W	3 mm
W0	10 mm
Thickness	0.3 mm

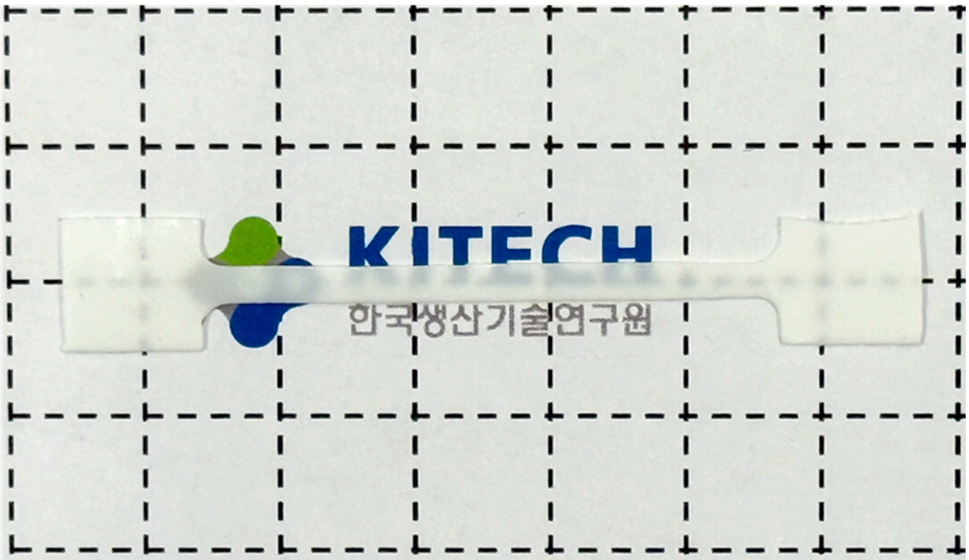

Figure S7. Dimensions and visual appearance of the dogbone-shaped specimen used for mechanical property measurements.
